# 

**Published:** 1882-03

**Authors:** 


					﻿Cn<A*5ORATORS:
ALF. L. LOOMIS; M. D......New York. I
FESSENDEN N. OTIS, M.
F. H. BOSWORTH, M.D........
J. L, LITTLE, M. I).......
C.F.. FRANCIS, D.D.S., M.D S.	•<'	,
A. J. C. SKENE, M. D.. Brooklyn,N . V. I
C. A. MARVIN, D. D. S..	“
W. C. BARRET!, M. D., D. D.S.. .Buffalo, “
EDWIN T. DARBY, M.D..D.D.S... Phil., Pa.
P. S. WALES, M. D„ Surg. Gen’l. U. S. Navy.
SAM’LU. BUSEV, M. D....Wash’n,	D. C.
H. E. CAMPBELL, M. D.....Augusta, Ga
C. H. MASTIN, M. I)., L.L.D.Mobile, Ala;
J. J. CHISOLM, M. D.. ....Baltimore, Md
C. F. BEVAN, M. D......... “
I.	E. ATKINSON, M.D....... “
H. McGUIRE, M. D.........Richmond, Va.
F. P. PORCHER, M. D.,....Charleston S. C.
J.	W. UNDERHILL, M. D .... Cincinnati, O.
IAS. T. WHITTAKER, M. D..
J. RANSOHOFF, M.D., F.R.C.S..	"
F. FORCHHEIMER, M. D.......	“	“
A. R. JACKSON, M. D........ Chicago, III
S. V. CLEVENGER, M.D.........“
E. F. INGALS, M. B......
J. H. CARSTENS, M.D.........Detroit, Mich.
WILL YOU PLEASE SEND IN YOUR SUBSCRIPTION MONEY NO W.
PROSPECTUS, 1882.
This Journal is cosmopolitan
in its circulation, management
and scientific resources, and of
equal interest to the specialist
and general practitioner
throughout the civilized world.
Its corps of editors and collab-
orators will represent Medicine,
Surgery, Obstetrics, Dentistry,
Hygiene and Popular Science,
and in these we will seek for that
which is new and useful, and
give the refined findings to our
readers. Politics, religious
creeds and isms, or preferences
in colleges and cliques will be
excluded, and, we will uphold,
truth and justice with indepen-
dence.
Please let us hear frbrn
you, and greatly oblige ''
Yours faithfully,
The Independent Practitioner Co.,
1099 BROADWAY, N. Y.
				

